# Suppression of Surface Waviness Error of Fresnel Micro-Structured Mold by Using Non-Integer Rotation Speed Ratio in Parallel Grinding Process

**DOI:** 10.3390/mi11070652

**Published:** 2020-06-30

**Authors:** Yongcheng Pan, Qingliang Zhao, Bing Guo, Bing Chen, Jinhu Wang

**Affiliations:** 1 Center for Precision Engineering, School of Mechatronics Engineering, Harbin Institute of Technology, Harbin 150001, China; panyongcheng19@126.com (Y.P.); zhaoqingliang@hit.edu.cn (Q.Z.); 2 Intelligent Manufacturing Institute, Hunan University of Science and Technology, Xiangtan 411201, China; chenbing@hnust.edu.cn; 3 Ultraprecision Machining Center, Zhejiang University of Technology, Hangzhou 310014, China; wangjinhu@zjust.edu.cn

**Keywords:** Fresnel micro-structured mold, surface waviness error, parallel grinding, non-integer rotation speed ratio, wave-shift value

## Abstract

Fresnel micro-structured lenses are widely used in the field of modern optoelectronic technology. High-precision Fresnel micro-structured mold is the key technology to achieve its large-scale replication production. Focusing on the surface waviness error of Fresnel micro-structured mold machined by parallel grinding process, this paper conducted theoretical modeling and experiment research. Based on the grinding kinematics theory, the simulation models of the surface waviness topography and the circular waviness profiles of the ground Fresnel micro-structured mold were developed, considering the combined influence of the non-integer rotation speed ratio and other grinding parameters. A series of grinding experiments were carried out to verify the proposed simulation models. The influence of a non-integer rotation speed ratio and a wave-shift value upon the surface waviness error of the ground Fresnel micro-structured molds were analyzed. Both the simulation and experimental results proved that choosing the non-integer rotation speed ratio and a proper wave-shift value could greatly reduce the surface waviness error and improve the surface quality and uniformity.

## 1. Introduction

Axisymmetric complex optical elements represented by Fresnel micro-structured lenses are widely used in modern optoelectronic technology [1,2]. Fresnel micro-structured lenses retain the refractive function of traditional convex/concave lenses, but reduce the thickness, volume and weight [3,4]. Fresnel lenses are widely used in projection display [5], photography [6], lighting optics [7], and solar energy [8]. In order to achieve large-scale and low-cost production, it is currently vigorously developing replication processing technologies such as glass molding [9,10,11,12], whose key component is Fresnel micro-structured mold. The mold material used for glass molding is mainly hard-brittle material such as silicon carbide (SiC) and tungsten carbide (WC) [13,14,15]. Ultra-precision grinding is the preferred manufacturing technology to achieve the high-precision requirements for hard-brittle components [16,17,18,19,20,21].

The surface quality evaluation parameters of optical elements include the form error of low frequency, the surface roughness of high frequency and the surface waviness error of intermediate frequency [22,23,24]. The periodic waviness error on the surface of the optical lens will cause reflection and diffraction, affecting the beam focusing performance [25]. In ultra-precision turning, the periodic characteristics of the surface waviness error are closely related to the vibration frequency of the tool and the workpiece [26,27]. Similarly, the surface waviness error is related to the rotation frequency of the workpiece and the grinding wheel in ultra-precision grinding process [28]. Therefore, the ratio of the grinding wheel’s rotation speed to the workpiece’s rotation speed is closely related to the generation of the surface waviness error of the ground workpiece, which is defined as the rotation speed ratio (RSR) [29]. RSR might be integer or non-integer, depending on the selection of the rotation speed of the grinding wheel and the workpiece. When the RSR is not integer, the fractional part of the non-integer RSR is defined as the wave-shift [30].

Due to unstable factors such as the grinding wheel’s run-out error, there often exists surface waviness error on the ground surface of lens molds [31,32], which is difficult to completely eliminate through a subsequent polishing process [33,34]. The surface waviness error will deteriorate the surface quality of the molds and affect the optical performance of the replicated Fresnel micro-structured lens. Compared with the research on form error and surface roughness of optical lens, there are few reports on the surface waviness error. Badger [35] proposed that the main source for the waviness error of the ground surface is the run-out error of the grinding wheel. Yoshihara [36,37] analyzed the influence of RSR on the grinding mark pattern and the nano topography of ground aspheric lenses. Chen Shanshan [38,39] suggested the method of continuously changing the workpiece rotation speed to suppress the surface waviness error and improve the surface uniformity in a parallel grinding process. In order to achieve the same material removal rate and uniform surface quality on all the circle surfaces of Fresnel micro-structured mold, Suzuki [40] proposed to use a variable feed rate to grind different circle surfaces. Based on the simulation of the distribution of grinding points, Chen Bing [41] proposed a prediction model for the grinding marks pattern of spherical lens and suggested that using proper non-integer RSR could improve the surface quality in a cross-grinding process. Pei [42,43] analyzed the influence of RSR on surface waviness error in the grinding process of silicon wafers. Tong [44] analyzed the relationship between RSR and the periodic distribution of the grinding scratch marks on ground lens surface. Trmal [30] investigated the surface waviness suppression effect of non-integer RSR in a cylindrical grinding process and pointed out the necessity of considering non-integer RSR and wave-shift when analyzing the influence of grinding parameters upon the surface quality of the workpiece. If ignoring them, some misleading conclusions might be obtained. Fricker [45] suggested that the surface waviness error was sensitive to the small changes of wave-shift in the cylindrical grinding process.

Based on the current reported research, it could be found that most of the research works on ground surface waviness error were concentrated on the waviness pattern. The qualitative correlation between waviness characteristics and grinding parameters was still unclear. Based on the grinding kinematics theory, through simulation and experiment methods, this paper studied the generation mechanism and influence factors of the surface waviness error of ground Fresnel micro-structured mold and proposed the method to suppress the surface waviness error by using non-integer RSR.

## 2. Development of Surface Waviness Model

### 2.1. Kinematical Analysis of the Parallel Grinding Process

**Figure 1:**
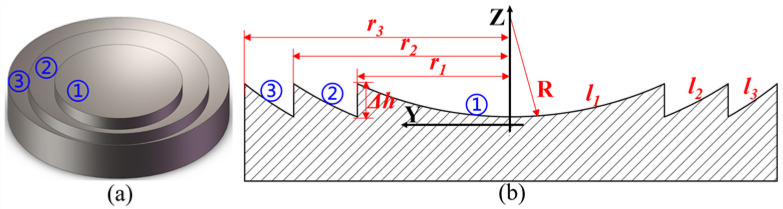
Fresnel micro-structured mold: (**a**) the 3D model; (**b**) the cross-section view.

**Figure 2:**
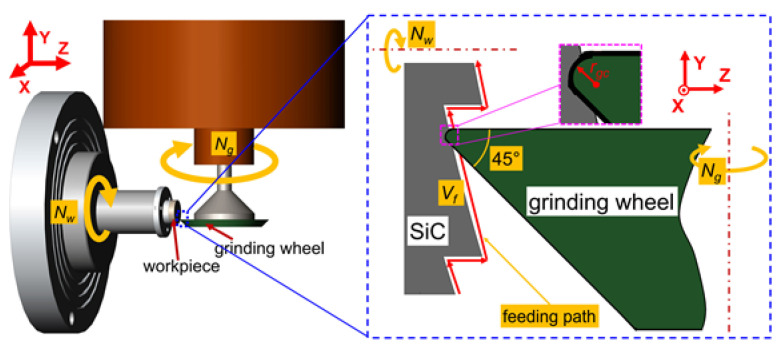
The grinding device scheme for the Fresnel micro-structured mold.

A typical spherical Fresnel micro-structured mold is shown in Figure 1, whose key feature dimensions include the curvature radius *R*, step height Δ*h* and the total number of circle surfaces *b*. The cross-section profile of the Fresnel micro-structured mold can be mathematically expressed by Equation (1). From the center to the edge of Fresnel micro-structured mold, the circle surface number is 1, 2… *b* respectively. The caliber radius *r_i_* of the *i*-th circle surface can be calculated by Equation (2). In order to realize the ultra-precision grinding of Fresnel micro-structured mold, the parallel grinding mode is chosen and a diamond grinding wheel with a sharp edge is used, as shown in Figure 2. A parallel grinding mode has been widely used for the machining of axisymmetric elements [46,47,48,49]. During the parallel grinding process, the grinding wheel and the workpiece rotate at a high speed and low speed, respectively. Through the simultaneous movement of two linear axes, e.g., *Y* and *Z*, the grinding wheel continuously feeds from the edge to the center of the workpiece along the planned path. The linear velocity direction of the rotational workpiece and the grinding wheel are parallel at the grinding point [48,49]:

(1) $$\begin{array}{ccc} Z\left(Y\right)=R-\left(i-1\right)\cdot \Delta h-\sqrt{R^{2}-Y^{2}} & & \left(r_{i-1}<\left|Y\right|<r_{i}\right) \end{array}$$

(2) $$\begin{array}{ccc} r_{i}=\sqrt{i\Delta h\left(2R-i\Delta h\right)} & & \left(i=1,2\ldots \ldots b\right) \end{array}$$

**Figure 3:**
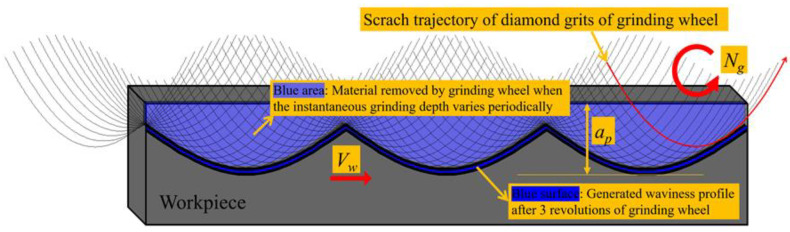
Generation mechanism of the surface waviness in the grinding process.

**Figure 4:**
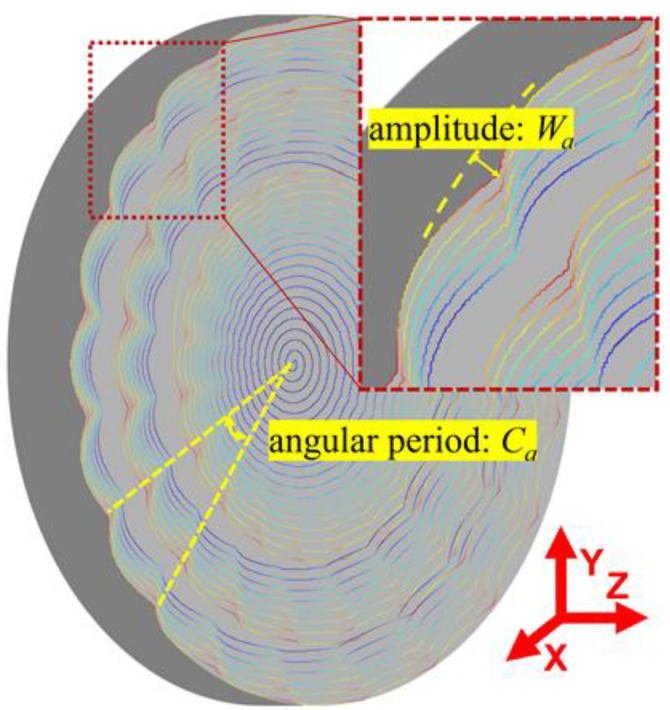
Surface waviness features of the ground Fresnel micro-structured mold.

Due to factors such as the random protrusion height of diamond grits, the uneven wear of the grinding wheel and the vibration of the grinding spindle, a certain amount of radial run-out error often exists on the grinding wheel. During the cycles of the grinding wheel’s revolutions, the grits’ instantaneous grinding depth varies periodically because of the radial run-out error, and then the periodically fluctuated surface waviness will be generated on the workpiece along the workpiece linear speed direction, as shown in Figure 3. Obviously, the surface waviness amplitude *W_a_* will not be higher than the grinding wheel’s run-out error *g_e_*. Since the tool path of the grinding wheel is a spiral line in a parallel grinding process, the surface waviness appearing on the Fresnel micro-structured mold distributes along its circular direction, as shown in Figure 4. From the edge to the center of the mold, the surface waviness amplitude *W_a_* decreases gradually because of the decreasing workpiece linear speed. Moreover, the angular period *C_a_* is used to characterize the period of the surface waviness.

**Figure 5:**
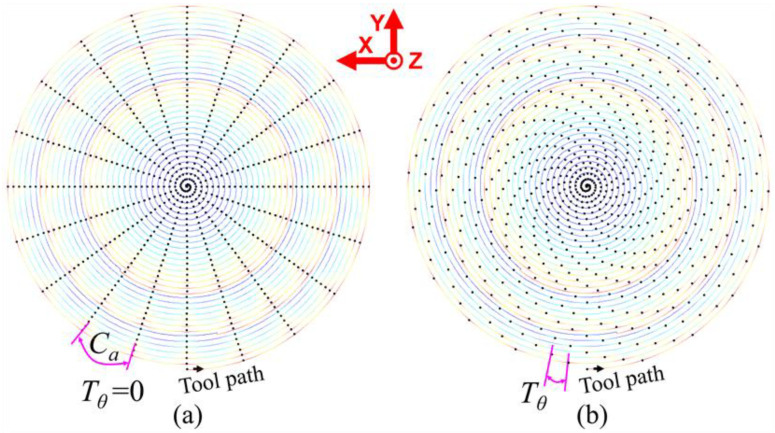
Grinding tool path and the wave-shift value of the Fresnel micro-structured mold: (**a**) the integer rotation speed ratio (RSR); (**b**) the non-integer RSR.

The rotation speed ratio (RSR) is an important parameter influencing the surface waviness features in the parallel grinding process, which equals the ratio of the grinding wheel’s rotation speed *N_g_* and the workpiece’s rotation speed *N_w_*, as described in Equation (3) [31]. When RSR is integer, the peaks and valleys of the circular waviness profiles generated during all the revolutions of the workpiece appear at the same angular position, thus forming a surface waviness pattern like a central radiant scallop, as shown in Figure 5a. However, when the RSR is non-integer, the generated surface waviness pattern will become a twisted scallop, as shown in Figure 5b. The fractional part of the non-integer RSR is defined as the wave-shift value *T_θ_*, which can be calculated by Equation (4) [31] and equals 0 when the RSR is integer. The function *round*(*R_N_*) means the nearest integer of *R_N_*:

(3) $$R_{N}=N_{g}/N_{w}$$

(4) $$T_{\theta }=\left|R_{N}-round\left(R_{N}\right)\right|\;\;\;\;\;\left(0\leq T_{\theta }\leq 1/2\right)$$

### 2.2. Modeling of the Surface Waviness Topography

**Figure 6:**
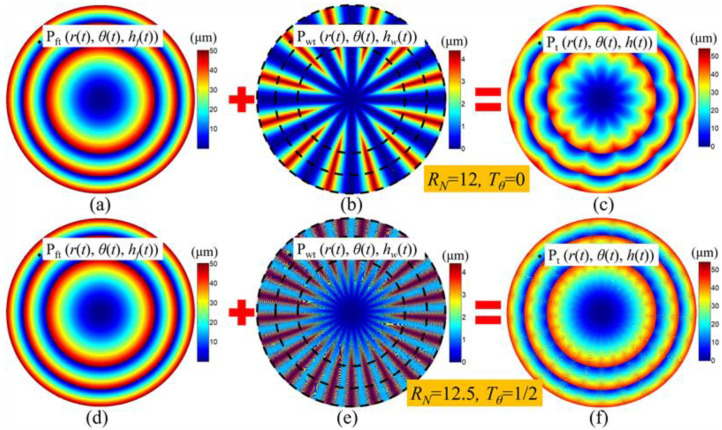
Simulation model of the surface waviness topography of the ground Fresnel micro-structured mold: (**a**,**d**) the ideal 3D model of the Fresnel mold; (**b**,**e**) the surface waviness model; (**c**,**f**) the 3D surface waviness topography of the Fresnel micro-structured mold.

During the parallel grinding process of the Fresnel micro-structured mold, because of the variable material removal rate from the edge to the center of workpiece, the generated surface waviness topography will be influenced by several grinding parameters. According to the grinding kinematics relationship, on the basis of the surface waviness simulation model [31] of the flat workpiece, a simulation model for the surface waviness topography of the Fresnel micro-structured mold can be established by using a cylindrical coordinate system, as shown in Figure 6. On the surface waviness topography of the Fresnel micro-structured mold, the grinding point *P_t_* (*r*(*t*), *θ*(*t*), *h*(*t*)) generated at time *t*, can be calculated by Equations (5)–(10), whose details are as follows:

(5) $$P\left(r\left(t\right),\theta \left(t\right),h\left(t\right)\right)=\{\begin{array}{lll} r\left(t\right)=R\cdot \sin\left(\arcsin\left(\frac{r_{b}}{R}\right)-\frac{V_{f}\cdot t}{R}\right) & & \\ \theta \left(t\right)=2\pi t\cdot N_{w} & & \left(0\leq t\leq t_{wa}\right)\\ h\left(t\right)=h_{f}\left(t\right)+h_{w}\left(t\right) & & \end{array}$$

(6) $$t_{wa}=\frac{R\cdot \arcsin\left(r_{b}/R\right)}{V_{f}}$$

(7) $$\begin{array}{ccc} h_{f}\left(t\right)=R-\left(i-1\right)\cdot \Delta h-\sqrt{R^{2}-r^{2}\left(t\right)} & & \left(r_{i-1}<r\left(t\right)<r_{i}\right) \end{array}$$

(8) $$h_{w}\left(t\right)=W_{a}\left(t\right)\cdot \left(1-\left|\cos\left(\pi t\cdot N_{g}\right)\right|\right)$$

(9) $$W_{a}\left(t\right)=\{\begin{array}{ll} \mathrm{if} & r_{b}\leq r_{wc}\\ & W_{a}\left(t\right)=0.5\cdot g_{e}\cdot \left(1-\cos\left(\pi \cdot r\left(t\right)/r_{wc}\right)\right)\;\;\;\;\;\left(0\leq r\left(t\right)\leq r_{b}\right)\\ \mathrm{else} & r_{b}>r_{wc}\\ & W_{a}(t)=\{\begin{array}{ccc} g_{e} & & \left(r_{wc}\leq r\left(t\right)\leq r_{b}\right)\\ 0.5\cdot g_{e}\cdot \left(1-\cos\left(\pi r\left(t\right)/r_{wc}\right)\right) & & \left(0\leq r\left(t\right)<r_{wc}\right) \end{array} \end{array}$$

(10) $$r_{wc}=(N_{g}/N_{w})\cdot \sqrt{0.5g_{e}\cdot r_{g}}$$

When comparing the simulation waviness topography of Fresnel micro-structured mold in Figure 6b,c and Figure 6e,f, which are machined by using integer RSR and non-integer RSR, respectively, it can be found that the surface quality and uniformity in Figure 6e,f are much better than in Figure 6b,c. Besides, the number of waviness cycles increases from 12 to 25 and the waviness amplitude decreases notably when using the non-integer RSR. In the next section, the suppression mechanism of the surface waviness error of the non-integer RSR will be discussed and the quantitative relationship between the surface waviness features and non-integer RSR will be analyzed.

### 2.3. Modeling of the Circular Waviness Profiles

**Figure 7:**
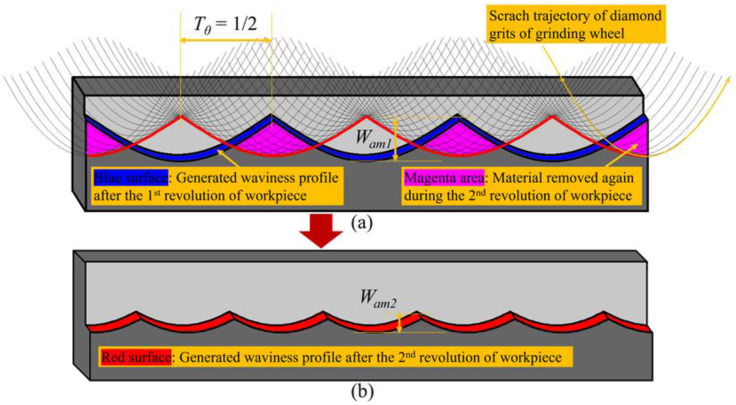
Suppression mechanism of the surface waviness amplitude when using non-integer RSR. (**a**) removing material again by the secondary grinding zone during the 2nd revolution of workpiece; (**b**) generated waviness profile after the 2nd revolution of workpiece.

In Figure 7, taking the condition when the wave-shift value equals $\frac{1}{2}$ as an example, the generation procedure of the surface waviness profile is described, which can explain the suppression mechanism of the surface waviness error of non-integer RSR. In a parallel grinding process, one thing to note [35] is that *l_g_* (the contact width between the grinding wheel and the workpiece) is much wider than *f_pr_* (the cross-feeding distance per revolution of workpiece). Therefore, the grinding wheel’s secondary grinding zone will remove the material again from the peak area of the just generated waviness profile, as shown in Figure 7a. After the material as gone through the primary and secondary grinding zone of the grinding wheel, the amplitude of the waviness profile will decrease and the finally generated waviness profile is the interference-overlapping result of the adjacent two waviness profiles, as shown in Figure 7b. Similarly, when the wave-shift value equals an Egypt fraction $\frac{1}{q}$, the finally generated waviness profile will be the interference-overlapping result of the adjacent *q* waviness profiles. However, there is an upper limit on the adjacent waviness profiles that can interfere and overlap, which is dependent on the ratio between *l_g_* and *f_pr_*.

**Figure 8:**
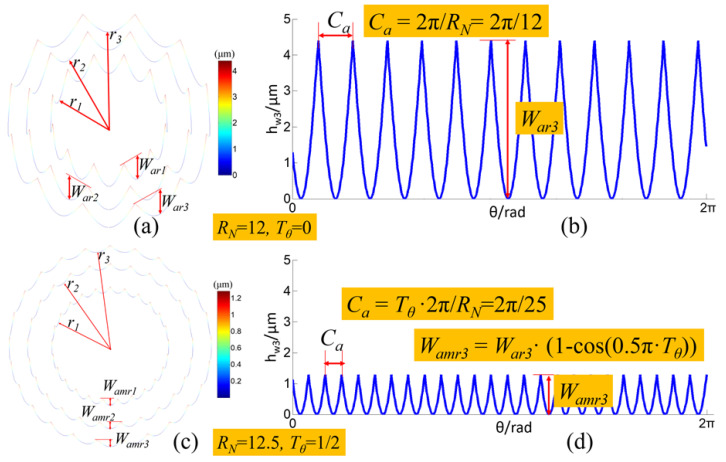
Simulation of the circular waviness profiles on the edge of each circle surface of the Fresnel micro-structured mold based on the waviness interference-overlapping effect. (**a**) circular waviness profiles of integer RSR; (**b**) horizontal expansion of the outermost circular waviness profile of integer RSR; (**c**) circular waviness profiles of non-integer RSR; (**d**) horizontal expansion of the outermost circular waviness profile of non-integer RSR.

As for the Fresnel micro-structured mold with several circle surfaces, the circular waviness profiles on the edge of each circle surface can be used to characterize its surface waviness distribution condition. When an RSR is integer, the circular waviness profiles can be mathematically described by Equation (11), whose period and amplitude can be calculated by Equations (12) and (13). When RSR is non-integer and the wave-shift value *T_θ_* = $\frac{1}{q}$, the circular waviness profiles can be mathematically described by Equation (14), whose period and amplitude can be calculated by Equations (15) and (16). Based on the Equations (11)–(16), the circular waviness profiles on the edge of each circle surface of the Fresnel micro-structured mold can be illustrated, as shown in Figure 8a,b and Figure 8c,d, representing the integer RSR and non-integer RSR, respectively.

(1) When RSR *R_N_* is integer and the wave-shift value *T_θ_* = 0, the circular waviness profile on the edge of the *i*-th circle surface of Fresnel micro-structured mold is:
  (11) $$\begin{array}{ccc} h_{wi}\left(\theta \right)=W_{ari}\cdot \left(1-\left|\cos\left(0.5\theta \cdot R_{N}\right)\right|\right) & & \left(0\leq \theta \leq 2\pi ;1\leq i\leq b\right) \end{array}$$
  (12) $$\mathrm{period}\;\mathrm{of}\;\mathrm{the}\;\mathrm{circular}\;\mathrm{waviness}\;\mathrm{profile}:C_{a}=\frac{2\pi }{R_{N}}$$
  (13) $$\begin{array}{c} \mathrm{amplitude}\;\mathrm{of}\;\mathrm{the}\;\mathrm{circular}\;\mathrm{waviness}\;\mathrm{profile}:\\ W_{ari}=\{\begin{array}{lll} \mathrm{if} & r_{i}\leq r_{wc} & \\ & W_{ari}=0.5g_{e}\cdot \left(1-\cos\left(\pi \cdot r_{i}/r_{wc}\right)\right) & \\ & & \left(1\leq i\leq b\right)\\ \mathrm{else} & r_{i}>r_{wc} & \\ & W_{ari}=g_{e} & \end{array} \end{array}$$
(2) When RSR *R_N_* is non-integer and the wave-shift value *T_θ_* = $\frac{1}{q}$ (*q* is integer, 2 ≤ *q* ≤ *round*(*l_g_*/*f_pr_*)), the circular waviness profile on the edge of the *i*-th circle surface of the Fresnel micro-structured mold:
  (14) $$\begin{array}{ccc} h_{wi}\left(\theta \right)=W_{ari}\cdot \left(1-\cos\left(0.5\theta \cdot R_{N}-\pi k\cdot T_{\theta }\right)\right) & & \left(\frac{\left(2k-1\right)\pi T_{\theta }}{R_{N}}\leq \theta \leq \frac{\left(2k+1\right)\pi T_{\theta }}{R_{N}};1\leq k\leq \frac{R_{N}}{T_{\theta }}\right) \end{array}$$
  (15) $$\mathrm{period}\;\mathrm{of}\;\mathrm{the}\;\mathrm{circular}\;\mathrm{waviness}\;\mathrm{profile}:C_{a}=\frac{2\pi T_{\theta }}{R_{N}}$$
  amplitude of the circular waviness profile:
  (16) $$W_{amri}=h_{wi}{\left(\theta \right)}_{max}-h_{wi}{\left(\theta \right)}_{min}=W_{ari}\cdot \left(1-\cos\left(0.5\pi \cdot T_{\theta }\right)\right)$$

## 3. Experiment and Simulation Results

### 3.1. Experimental Setup and Parameters Design

**Figure 9:**
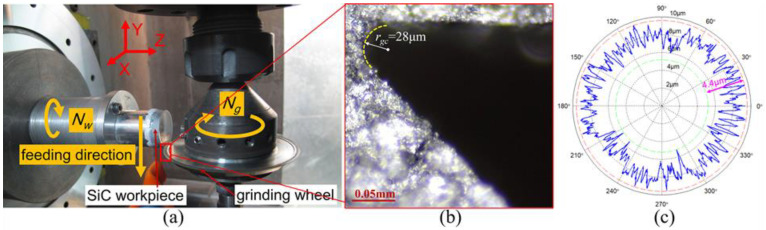
The grinding experiment system for the Fresnel micro-structured mold: (**a**) the grinding experiment devices; (**b**) the copied profile of the edge tip of the grinding wheel; (**c**) the measurement result of the radial run-out error of the grinding wheel

In order to verify the proposed simulation models of the surface waviness topography and the circular waviness profile of the Fresnel micro-structured mold, a series of grinding experiments were carried out. The grinding experiment system was built on a four-axis ultra-precision grinding machine, as shown in Figure 9a. The specific experiment condition was listed in Table 1. Before the grinding process, the upper face and the hypotenuse face of the grinding wheel were conditioned precisely by using a green silicon carbide rod [50,51]. After the conditioning procedure, the edge tip profile of the grinding wheel was copied onto a graphite block and captured by a digital microscope (850×), as shown in Figure 9b. In addition, a laser displacement sensor was utilized to measure the radial run-out error of the grinding wheel and the measurement result is shown in Figure 9c.

**Figure 10:**
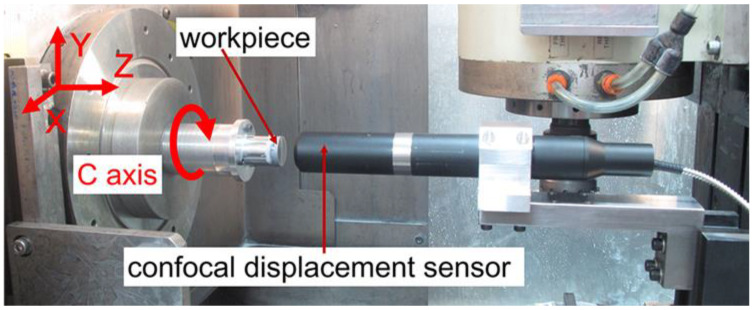
The on-machine measurement devices of the circular waviness profile of the Fresnel micro-structured mold.

In order to study the influence of non-integer RSR on the surface waviness of the ground Fresnel micro-structured mold, the grinding wheel’s rotation speed was slightly varied to achieve a different wave-shift value, but other parameters were kept constant, as listed in Table 2. After the grinding process, the photos of these ground Fresnel micro-structured molds were captured by another digital microscope (14×). Moreover, the circular profile at the edge of each circle surface of the Fresnel micro-structured mold was measured on-machine by using a chromatic confocal displacement sensor with the C axis mode of the machine tool, as shown in Figure 10.

### 3.2. Analysis of Experiment and Simulation Results

**Figure 11:**
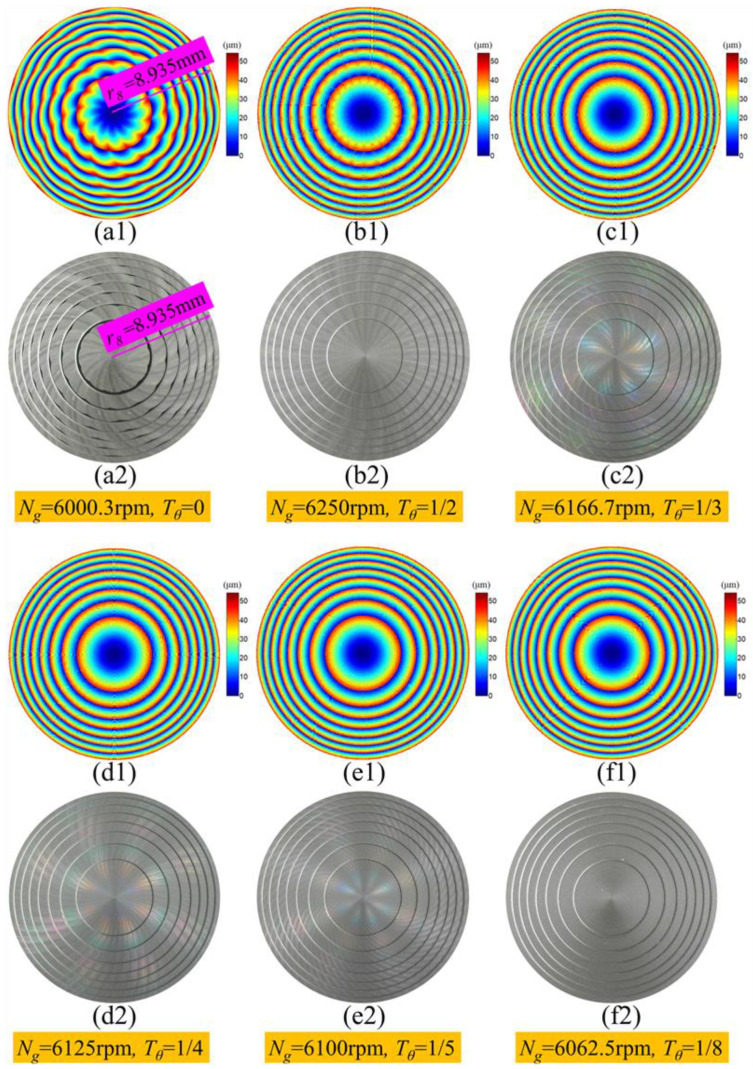
Simulation and the experiment result of the surface waviness topography of the ground Fresnel micro-structured molds: (**a1**–**f1**) the simulation results; (**a2**–**f2**) the photos of the workpiece.

Figure 11a1–f1 and Figure 11a2–f2 show the simulation surface waviness topography and the photos of the Fresnel micro-structured molds machined with different wave-shift values, respectively. In general, the simulation results matched well with the experimental results. Both the simulation and experiment results revealed that the surface waviness error was weaker and the surface quality was smoother by using non-integer RSR compared to integer RSR. Among these ground Fresnel micro-structured molds, the surface quality was roughest when *T_θ_* = 0 and smoothest when *T_θ_* = $\frac{1}{8}$. When the wave-shift value *T_θ_* decreased gradually from $\frac{1}{2}$ to $\frac{1}{8}$, the surface uniformity was improved and the surface waviness error was suppressed gradually. Therefore, the proposed simulation model of the surface waviness topography was proved to be correct. It should be pointed out that the simulation waviness topography in Figure 11c2–f2 was not clear and obvious when the wave-shift value *T_θ_* equaled,$\frac{1}{3}$, $\frac{1}{4}$, $\frac{1}{5}$ and $\frac{1}{8}$, because the amplitude of the suppressed surface waviness was too low compared to the step height of the Fresnel micro-structured mold.

**Figure 12:**
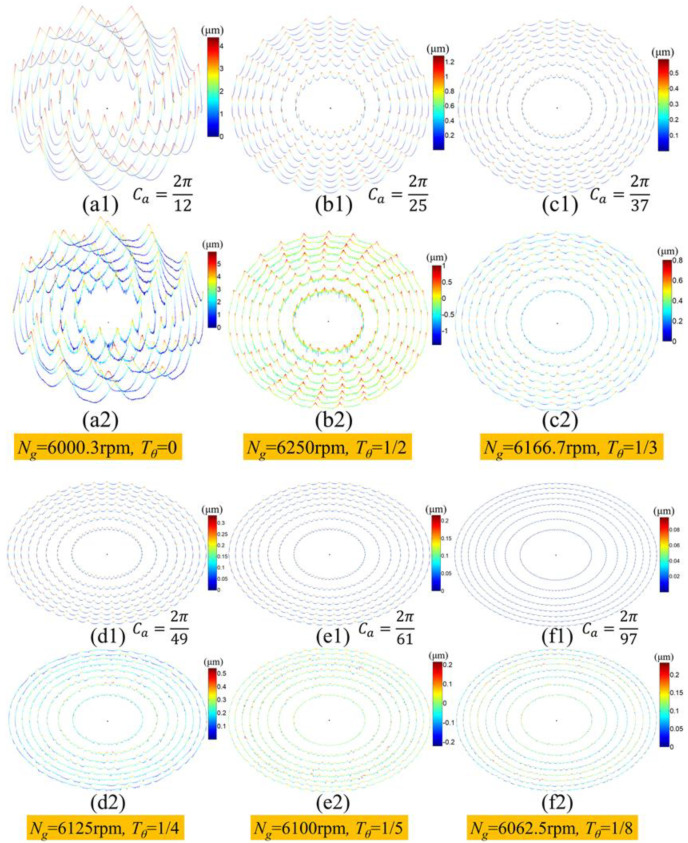
Simulation and the measurement results of the circular waviness profile on each circle surface of the Fresnel micro-structured molds: (**a1**–**f1**) the simulation results; (**a2**–**f2**) the measurement results.

Figure 12a1–f1 and Figure 12a2–f2 show the simulation and measurement results, respectively, of the circular waviness profiles at the edge of each circle surface of Fresnel micro-structured mold, which were machined with different wave-shift values. The specific amplitude values of the simulation and measurement circular waviness are listed in Table 3. It was obvious that the simulation profiles matched well with the measurement profiles. Overall, the angular period and amplitude of the waviness profiles was the highest when *T_θ_* = 0. With the wave-shift value *T_θ_* decreased gradually from $\frac{1}{2}$ to $\frac{1}{8}$, the number of waviness cycles increased, but the amplitude and angular period declined, thus improving the surface quality and uniformity of the ground Fresnel micro-structured mold. Therefore, the proposed simulation model of the circular waviness profile was proved to be correct. It should be pointed out that the simulation profiles were not perfectly similar to the measurement profiles when the wave-shift value *T_θ_* equaled $\frac{1}{4}$, $\frac{1}{5}$ and $\frac{1}{8}$, due to the random deviation of the grinding spindle’s rotation speed and imperfect interference-overlapping effect of adjacent waviness profiles.

**Figure 13:**
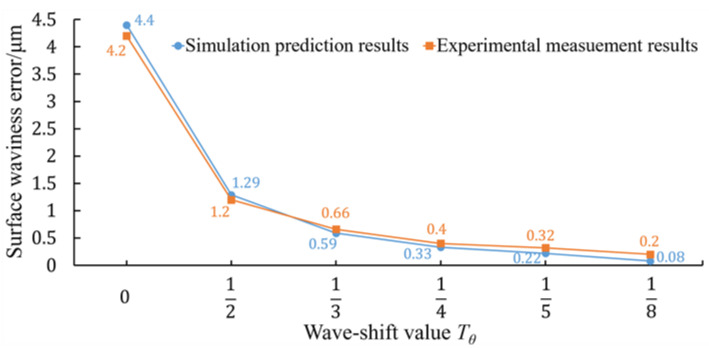
Simulation and measurement results of the surface waviness error of ground Fresnel micro-structured molds.

The maximum of the amplitude of the circular waviness profiles of the Fresnel micro-structured mold was used to evaluate its surface waviness error. The varying trend of the surface waviness error of the Fresnel micro-structured mold machined with different wave-shift values is illustrated in Figure 13. It could be found that the simulation results have the same trend as the experimental results, although there existed a small deviation between the two data lines. When the RSR was non-integer, the amplitude decreased gradually with the decrease in the wave-shift value. The experimental results showed that the surface waviness error could be reduced from 4.2 μm to 0.2 μm when adjusting the wave-shift value from 0 to $\frac{1}{8}$, whose drop rate reached 95%.

**Figure 14:**
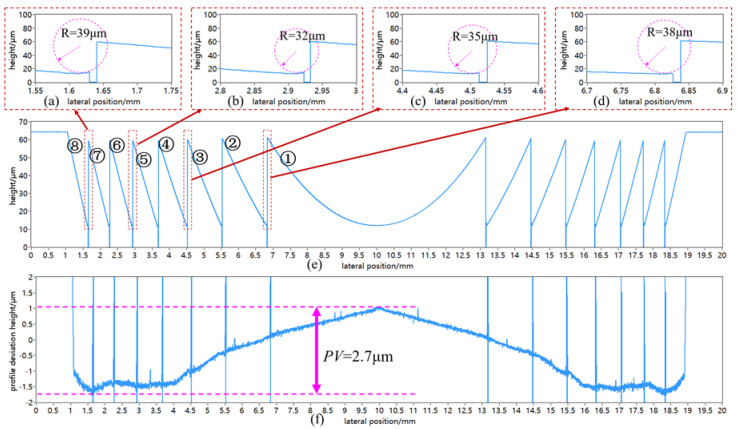
Analysis of the measured cross-section profile of the ground Fresnel micro-structured mold by a chromatic confocal displacement sensor. (**a**) evaluation of the corner radius of the 7th valley corner; (**b**) evaluation of the corner radius of the 5th valley corner; (**c**) evaluation of the corner radius of the 3rd valley corner; (**d**) evaluation of the corner radius of the 1st valley corner; (**e**) measurement result of the cross-section profile of the ground Fresnel micro-structured mold; (**f**) profile deviation analysis of the ground Fresnel micro-structured.

The cross-section profile of the Fresnel micro-structured mold machined by using *T_θ_* = $\frac{1}{8}$ was measured and analyzed, as shown in Figure 14. It is noteworthy that since the vertical side of the valley corner was too steep, no reflected light signal could be received by the chromatic confocal displacement sensor and the output measurement data would be zero. The profile deviation was obtained by calculating the deference between the measurement Fresnel profile and the theoretical Fresnel profile, as illustrated in Figure 14f, the form error *PV* = 2.7 μm. Moreover, thanks to the suppression effect of surface waviness, the microscopic surface fluctuations in Figure 14f along the diameter of the ground Fresnel micro-structured mold were basically uniform and stable, except for the valley corner area between two adjacent circle surfaces. The valley corner area was affected by the grinding wheel’s edge radius (28 μm), thus generating a curvature. The corner radius was evaluated through the circle-fitting method, as shown in Figure 14a–d. The evaluation results indicate that the corner radius was about 32–39 μm, which was close to the grinding wheel’s edge radius (28 μm), but slightly larger. The reason might be the random distribution of abrasive grits at the edge of the grinding wheel, or measurement errors. To sum up, the measurement results of the circular waviness profiles and the cross-section profile both proved that using the non-integer RSR and proper wave-shift value could significantly suppress the surface waviness error and improve the surface quality and uniformity of the Fresnel micro-structured mold in a parallel grinding process.

## 4. Conclusions

Focusing on the surface waviness error of the Fresnel micro-structured mold machined by the parallel grinding process, this paper conducted theoretical modeling and experimental research and some conclusions could be drawn.

(1) Based on the surface waviness simulation model of the ground plane workpiece and the grinding kinematics theory, the simulation model of the surface waviness topography of the ground Fresnel micro-structured mold was developed and experimentally verified. As a typical example, it could provide a solution to help extend the ground surface waviness simulation model to other complexed axisymmetric components, such as aspheric lens, ball valve and cylindrical components.
(2) Based on the interference-overlapping effect of the adjacent waviness profiles, the theoretical simulation model of the circular waviness profiles of the ground Fresnel micro-structured mold was firstly developed and experimentally verified. Compared with the simulated surface waviness topography, it could more clearly and three-dimensionally illustrate the distribution of waviness error at a different radial position of the ground workpiece surface, and more distinctly show the suppression effect of the surface waviness error by using different wave-shift values. The experimental results showed that the surface waviness error could be reduced by 95%, when slightly adjusting the grinding wheel’s rotation speed to achieve a proper wave-shift value.
(3) The proposed simulation model of the surface waviness topography and circular waviness profiles could be used to predict the surface waviness error of the ground Fresnel micro-structured mold and guide the selection of grinding parameters. Using non-integer RSR and a proper wave-shift value could significantly suppress the surface waviness error and improve the surface quality and uniformity in parallel to the grinding process of complexed axisymmetric components, without decreasing the production efficiency.

## Figures and Tables

**Table 1:** Grinding experiment conditions.

Item	Detail
Grinding wheel	Metal bonded diamond grinding wheel, WINTER^®^, BZ1V1-75-6.35-3-45 12.7*D15A BZ387-C50, diameter 75 mm, grits size 8–15 μm, tip nose radius 28 μm
Workpiece	Pressureless sintering silicon carbide (SiC), Hexoloy^®^ SA, grain size 4–10 μm, diameter 20 mm, thickness 5 mm
Machine tool	4-axis ultra-precision grinding machine tool
Laser displacement sensor	KEYENCE^®^ H020, accuracy 0.1 μm
Chromatic confocal displacement sensor	STIL^®^ MG140, accuracy: 25 nm
Digital microscope	Dino-Lite^®^, AM7515MT8A (700×–900×), AM7115MZTL (10×–140×)
Fresnel micro-structured mold	Total number of circle surfaces *b* = 8; step height Δ*h* = 50 μm; spherical radius *R* = 100 mm
Rotation speed of workpiece (*N_w_*)	500 rpm
Feed rate (*V_f_*)	1 mm/min
Grinding depth (*a_p_*)	5 μm
Grinding fluid	Water based, challenge 300-HT, concentrations 2–3%

**Table 2:** Experiment parameters design.

No.	Rotation Speed of Grinding Wheel *N_g_* (rpm)	Rotation Speed Ratio *R_N_*	Wave-Shift Value *T_θ_*
1	6000.3	12	0
2	6250	12$\frac{1}{2}$	$\frac{1}{2}$
3	6166.7	12$\frac{1}{3}$	$\frac{1}{3}$
4	6125	12$\frac{1}{4}$	$\frac{1}{4}$
5	6100	12$\frac{1}{5}$	$\frac{1}{5}$
6	6062.5	12$\frac{1}{8}$	$\frac{1}{8}$

**Table 3:** Simulation and measurement results of the circular waviness amplitude on each circle surface of the Fresnel micro-structured molds.

Wave Shift Value	Circular Waviness Amplitude on the Edge of Each Circle Surface of Fresnel Micro-Structured Molds (μm)							
	Circle Surface ①*W_amr1_*	Circle Surface ②*W_amr2_*	Circle Surface ③*W_amr3_*	Circle Surface ④*W_amr4_*	Circle Surface ⑤*W_amr5_*	Circle Surface ⑥*W_amr6_*	Circle Surface ⑦*W_amr7_*	Circle Surface ⑧*W_amr8_*
*T_θ_* = 0 (simulation)	4.34	4.4	4.4	4.4	4.4	4.4	4.4	4.4
*T_θ_* = 0 (measurement)	3.57	3.71	3.77	4.05	4.12	4.04	4.14	4.2
*T_θ_* = $\frac{1}{2}$ (simulation)	1.25	1.29	1.29	1.29	1.29	1.29	1.29	1.29
*T_θ_* = $\frac{1}{2}$ (measurement)	0.85	0.95	1.04	0.98	1.01	1.07	1.15	1.2
*T_θ_* = $\frac{1}{3}$ (simulation)	0.57	0.59	0.59	0.59	0.59	0.59	0.59	0.59
*T_θ_* = $\frac{1}{3}$ (measurement)	0.46	0.51	0.55	0.57	0.56	0.59	0.65	0.66
*T_θ_* = $\frac{1}{4}$ (simulation)	0.32	0.33	0.33	0.33	0.33	0.33	0.33	0.33
*T_θ_* = $\frac{1}{4}$ (measurement)	0.31	0.37	0.34	0.35	0.32	0.31	0.4	0.4
*T_θ_* = $\frac{1}{5}$ (simulation)	0.21	0.22	0.22	0.22	0.22	0.22	0.22	0.22
*T_θ_* = $\frac{1}{5}$ (measurement)	0.26	0.29	0.31	0.32	0.31	0.32	0.3	0.32
*T_θ_* = $\frac{1}{8}$ (simulation)	0.08	0.08	0.08	0.08	0.08	0.08	0.08	0.08
*T_θ_* = $\frac{1}{8}$ (measurement)	0.11	0.12	0.16	0.18	0.12	0.13	0.2	0.2

## Abbreviations

*a_p_* (μm)	Grinding depth
*C_a_* (°)	Angular period value of the surface waviness of Fresnel micro-structured mold
*f_pr_* (mm)	Cross-feeding distance per revolution of workpiece
*g_e_* (mm)	Radial run-out error of grinding wheel
Δ*h* (μm)	Step height of Fresnel micro-structured mold
*l_g_* (mm)	Contact length between grinding wheel and workpiece
*b*	Total number of circle surfaces of Fresnel micro-structured mold
*N_g_* (rpm)	Rotation speed of grinding wheel
*N_w_* (rpm)	Rotation speed of workpiece
*R* (mm)	Spherical radius of Fresnel micro-structured mold
*R_N_*	Rotation speed ratio (RSR) of grinding wheel and workpiece
*r_g_* (mm)	Radius of grinding wheel
*r_gc_* (μm)	Tip nose radius of grinding wheel
*r_i_* (mm)	Caliber radius of the *i*-th circle surface of Fresnel micro-structured mold
*r_wc_* (mm)	Critical radial position on workpiece in parallel grinding process of Fresnel micro-structured mold
*T_θ_*	Wave-shift value
*t_wa_* (min)	The total machining time of a complete feeding procedure from the edge to the center of Fresnel micro-structured mold in parallel grinding process
*V_f_* (mm/min)	Feed rate of grinding wheel
*W_a_* (μm)	Amplitude of the surface waviness of Fresnel micro-structured mold
*W_amri_* (μm)	Amplitude of the surface waviness profile at the edge of the *i*-th circle surface of Fresnel micro-structured mold (non-integer RSR)
*W_ari_* (μm)	Amplitude value of the surface waviness profile at the edge of the *i*-th circle surface of Fresnel micro-structured mold (integer RSR)
*W_at_* (μm)	Amplitude of the generated surface waviness on Fresnel micro-structured mold at time *t* during the feeding procedure
